# UCO Physical Rehabilitation: New Dataset and Study of Human Pose Estimation Methods on Physical Rehabilitation Exercises

**DOI:** 10.3390/s23218862

**Published:** 2023-10-31

**Authors:** Rafael Aguilar-Ortega, Rafael Berral-Soler, Isabel Jiménez-Velasco, Francisco J. Romero-Ramírez, Manuel García-Marín, Jorge Zafra-Palma, Rafael Muñoz-Salinas, Rafael Medina-Carnicer, Manuel J. Marín-Jiménez

**Affiliations:** 1 Departamento de Informática y Análisis Numérico, Edificio Einstein, Campus de Rabanales, Universidad de Córdoba, 14071 Córdoba, Spain; raortega@uco.es (R.A.-O.); rberral@uco.es (R.B.-S.); isajimenez@uco.es (I.J.-V.); fj.romero@uco.es (F.J.R.-R.); rmsalinas@uco.es (R.M.-S.); rmedina@uco.es (R.M.-C.); 2 Departmento de Rehabilitación, Hospital Universitario de Jaén, Avenida del Ejército Español nº10, 23007 Jaén, Spain; manuel.garcia.ma.sspa@juntadeandalucia.es; 3 Instituto Maimónides de Investigación en Biomedicina (IMIBIC), Avenida Menéndez Pidal s/n, 14004 Córdoba, Spain; jzafra@uco.es

**Keywords:** human pose estimation, rehabilitation exercises, dataset, deep learning

## Abstract

Physical rehabilitation plays a crucial role in restoring motor function following injuries or surgeries. However, the challenge of overcrowded waiting lists often hampers doctors’ ability to monitor patients’ recovery progress in person. Deep Learning methods offer a solution by enabling doctors to optimize their time with each patient and distinguish between those requiring specific attention and those making positive progress. Doctors use the flexion angle of limbs as a cue to assess a patient’s mobility level during rehabilitation. From a Computer Vision perspective, this task can be framed as automatically estimating the pose of the target body limbs in an image. The objectives of this study can be summarized as follows: (i) evaluating and comparing multiple pose estimation methods; (ii) analyzing how the subject’s position and camera viewpoint impact the estimation; and (iii) determining whether 3D estimation methods are necessary or if 2D estimation suffices for this purpose. To conduct this technical study, and due to the limited availability of public datasets related to physical rehabilitation exercises, we introduced a new dataset featuring 27 individuals performing eight diverse physical rehabilitation exercises focusing on various limbs and body positions. Each exercise was recorded using five RGB cameras capturing different viewpoints of the person. An infrared tracking system named OptiTrack was utilized to establish the ground truth positions of the joints in the limbs under study. The results, supported by statistical tests, show that not all state-of-the-art pose estimators perform equally in the presented situations (e.g., patient lying on the stretcher vs. standing). Statistical differences exist between camera viewpoints, with the frontal view being the most convenient. Additionally, the study concludes that 2D pose estimators are adequate for estimating joint angles given the selected camera viewpoints.

## 1. Introduction

Physical rehabilitation is a common recovery method following body joint surgery (e.g., frozen shoulder, humeral and clavicle fractures, anterior cruciate ligament reconstruction, knee fractures, patellofemoral pain syndrome, and knee arthroplasty [1]). This method aims to achieve complete and satisfactory patient recovery and avoid further complications. About 90% of this rehabilitation procedure is performed by the patient at home without the supervision of a medical professional [2]. This can lead to a deficit in the consistency and quality of the exercises performed by patients.

Computer-guided methods for automatically analyzing the exercises performed by the patients during the rehabilitation period can help medical staff to evaluate the effectiveness of these treatments and the correct performance of patients. In addition, these methods can provide the necessary tools for the patient to carry out such rehabilitation exercises at home in a remote scenario.

These methods can be classified into those that require sensors or specific machinery (like wearables) [3] and Computer Vision-based methods, with less constraining requirements, often using the output of an RGB camera such as the ones in a modern smartphone. Computer Vision-based methods can be divided into two groups: based on human pose estimation (HPE) [4,5], where the position of each body joint is estimated, and based on activity recognition [6,7], where the recognition is driven by segmenting the position of the individuals or other objects in the scene. The use of cameras to analyze real-world physiotherapy is a challenging task but is less invasive than the use of wearable devices and allows patients to be independent of specialized and expensive devices.

The authors in [8] and [9] show that an accurate estimation of the human skeleton is a key step for the assessment of human motion, especially in the task of physical rehabilitation. Computer Vision methods based on Deep Learning are strongly dependent on training data. The amount and quality of training data are decisive for the quality of the result returned by the model. Although there are datasets dedicated to the task of physical rehabilitation of patients, most do not include RGB information [10,11]. They only attach pose information, depth maps, or other specific data about the performance of the exercises. These RGB data are crucial for the training of specific HPE methods for the rehabilitation task, fine tuning of existing methods, or feature extraction for analysis and assessment of the performed exercises.

Currently, studies on the use of systems to assist the rehabilitation of physical therapy patients using video cameras are a growing field of research. For example, the authors in [12] use a single-camera viewpoint to analyze gait metrics. Also in [13], the authors use cameras and pose estimation methods in order to classify different physical therapy exercises recorded using two smartphone cameras. Finally, we should mention that the study of the influence of camera angle for human pose estimation has also been studied in [14].

Our contribution is two-fold: (i) a new dataset, coined “UCO Physical Rehabilitation” (dataset available at https://github.com/AVAuco/ucophyrehab under request. Last access: 19 September 2023), consisting of 2160 videos showing people performing rehabilitation exercises, as the ones prescribed by physiotherapists after body joint surgery; and, (ii) a thorough evaluation of state-of-the-art Computer Vision-based methods for automatic human pose estimation in monocular images from three different points of view—(a) the accuracy of these methods in physical rehabilitation purposes, (b) the influence of the point of view on the estimation, and (c) the comparison of the use of 2D over 3D methods for this task.

**Figure 1:**
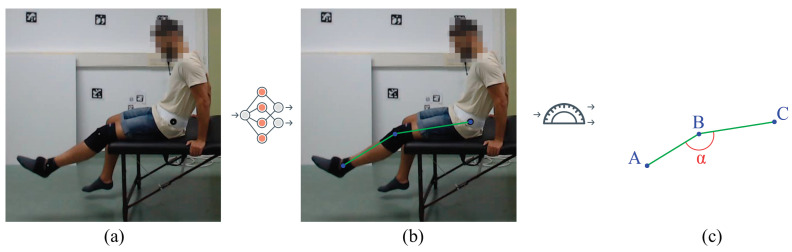
**Objective of this work.** The goal of this work is to experimentally evaluate the usability of general state-of-the-art HPE methods, on monocular images, in the context of physical rehabilitation. (**a**) Sample image from the new annotated dataset of physical rehabilitation exercise videos. (**b**) The video frames are processed with diverse state-of-the-art HPE methods, mostly based on neural networks, obtaining the location of the body joints per frame. (**c**) As an application, the estimated body joints can be used to automatically obtain rehabilitation metrics as the flexion angle of the leg (A = ankle, B = knee, C = hip).

We performed an experimental study to test the influence of camera angles and patient positions (by performing different exercises) on the detection accuracy of different state-of-the-art human pose estimation models. The experimental results show that most state-of-the-art methods work relatively well for upright positions but tend to fail when the person is lying on their back (i.e., supine position). Therefore, we carried out a comparative study on the influence of rotating some of the input videos to simulate the patient standing instead of lying on their back. The performance of the exercises was evaluated by calculating the angle of the involved body joint in the exercise type (see Figure 1).

The rest of this paper is structured as follows. Section 2 presents the new dataset UCO Physical Rehabilitation. Then, Section 3 describes the methods we use in Section 4, which shows the experiments. The results are presented in Section 5 and discussed in Section 6. Finally, in Section 7, we set out our conclusions and future work.

## 2. Materials: The UCO Physical Rehabilitation Dataset

The proposed dataset, coined “UCO Physical Rehabilitation”, was recorded from June to July 2022. A total of 27 healthy subjects (7 females and 20 males), aged 23 to 60 years, were recorded indoors in a controlled environment.

**Figure 2:**
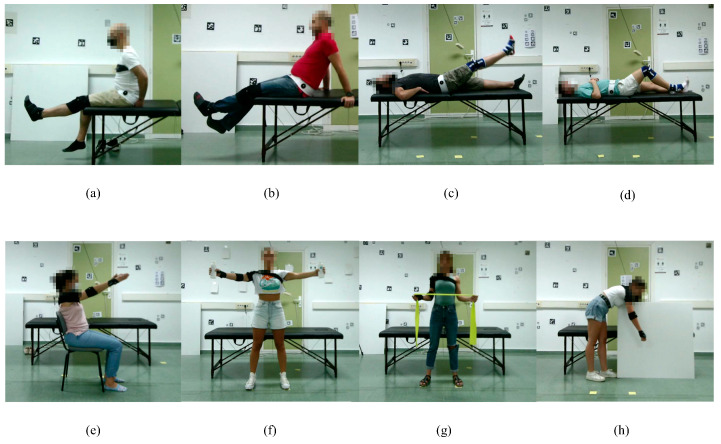
**Examples of some of the exercises included in the dataset**: (**a**) Exercise 01, (**b**) Exercise 02, (**c**) Exercise 03, (**d**) Exercise 04, (**e**) Exercise 13, (**f**) Exercise 14, (**g**) Exercise 15, (**h**) Exercise 16. All views from the viewpoint of Camera 2 (see Figure 3).

Subjects were instructed to perform four exercises for the lower body and four exercises for the upper body on both sides (left and right), obtaining a total of 16 different activities (see Figure 2). Each activity consists of four repetitions of the same exercise at a low speed to simulate a recent surgery or injury. A team of surgeons and physiotherapists provided the list of exercises, which are summarized in Table 1, classified by position, lower or upper body and side or orientation of the subject.

Hence, the dataset contains the following cases:

- Exercises 01 and 05—Starting in a seated position, raise the leg as high as possible.
- Exercises 02 and 06—In the same position as above, raise the leg as high as possible with the support of the other leg for raising and lowering.
- Exercises 03 and 07—Lying flat on the treatment couch, raise the leg straight up as high as possible.
- Exercises 04 and 08—In the same position as above, bend the knee with the heel on the treatment couch as much as possible.
- Exercises 09 and 13—Seated in a chair, raise both arms straight as high as possible.
- Exercises 10 and 14—Standing in front of the cameras, open and close both arms straight holding a light object.
- Exercises 11 and 15—Standing in the same place as above, with elbows close to the body, stretch the rubber band with shoulder rotation.
- Exercises 12 and 16—Standing in front of the treatment couch, with the other hand resting on the treatment couch, perform a pendulum shoulder rotation.

Overall, 2160 video sequences, with an average duration of 30.4 s (about 1.6M frames in total), were recorded with 5 RGB cameras of 1280×720 pixel resolution. Each camera recorded a different point of view of the scene.

**Figure 3:**
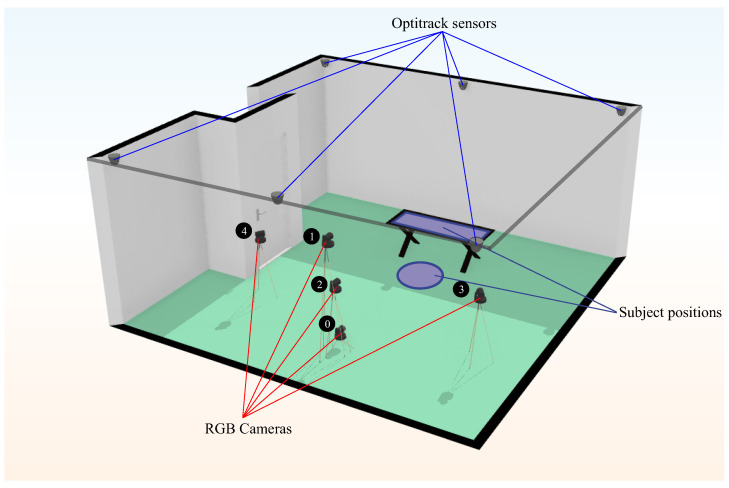
**Plan of the cameras in the laboratory**. The scheme shows both the location of the OptiTrack motion capture system (solid blue lines) and the RGB cameras (solid red lines). The positions of the subject are represented as a blue circle and a blue rectangle (over the treatment couch).

The cameras were placed at three different angles and three different heights. Figure 3 shows the camera setup. Cameras 0, 1 and 2 had the same horizontal angle with respect to the scene, but each one was placed at a different height. Camera 0 was 15 cm above the ground, and camera 1 was 180 cm. The rest of the cameras were placed 100 cm above the ground.

At the same time, the scene was recorded with an IR motion capture system (OptiTrack, see more at: https://optitrack.com/cameras/flex-3/. Last access: 19 September 2023) with six cameras placed in an elevated position, as we can see in Figure 3. Subjects were fitted with infrared sensors on the shoulder, elbow and wrist for upper body exercises and on the hip, knee and ankle for lower body exercises. These IR sensors provide the 3D ground truth points for each record on the dataset with a 3D accuracy of ±0.5 mm (Optitrack Flex 3 datasheet: https://d111srqycjesc9.cloudfront.net/Flex%203%20Data%20Sheet.pdf. Last access: 19 September 2023) each one. For each frame recorded, we stored five frames captured from the RGB cameras and the 3D ground truth points of the sensors returned by OptiTrack. Subsequently, we obtained the 2D ground truth points from these 3D ground truth points by projection.

Note that due to the color of the treatment couch and the individuals’ position, we had to place a white square item in the background for the last exercise (Exercises 12 and 16, see Figure 2h). OptiTrack could not obtain the ground truth for this particular exercise without that addition.

### Data Pre-Processing

While recording the videos, we noticed two problems with the IR motion capture system:

1. The order of the ground truth positions for the three joints changed from video to video and even from one frame to another. As such, it was difficult to know which point corresponded to each joint.
2. In some frames, not every ground truth value was recovered (i.e., there would be a missing joint).

To solve the first problem, we pre-processed the OptiTrack output in order to keep a fixed order in the 3D ground truth points and consequently in the 2D ground truth points obtained afterwards.

The order of the body joint points is as follows:

- For upper body exercises: shoulder (0), elbow (1) and wrist (2).
- For lower body exercises: hip (0), knee (1) and ankle (2).

To partially solve the second problem, we manually labeled the missing joints in those frames in 2D where they could not be detected. In this way, we were able to recover those frames and as many 2D keypoints as possible. It should be noted that these frames do not have any 3D ground truth point and, therefore, were not taken into account in the experiments we performed with the 3D ground truth points (see Section 4).

## 3. Methods

In this section, we introduce the methods considered for comparison in this work. Please do note that for each method, their training was carried out on general-purpose datasets; thus, they are not specifically aimed at physical rehabilitation tasks. Our experimental study tried to shed some light on whether such methods suit the task.

**Figure 4:**
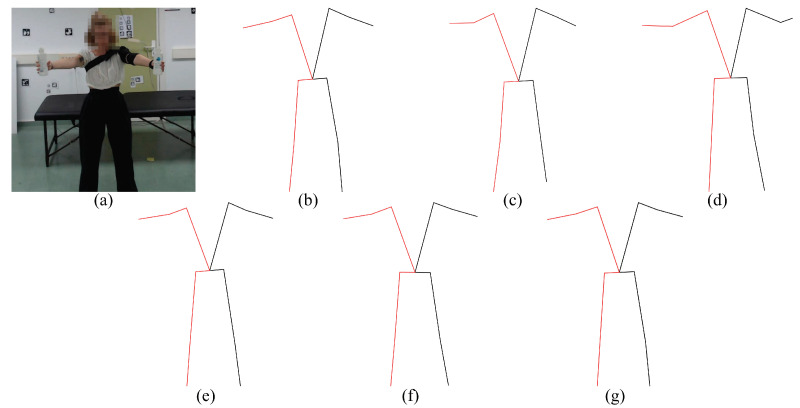
**Preview of the evaluated 2D pose estimation models**. (**a**) Input frame, (**b**) AlphaPose, (**c**) MediaPipe, (**d**) HMR, (**e**) VideoPose3D, (**f**) KAPAO, (**g**) StridedTransformer-Pose3D.

**Figure 5:**
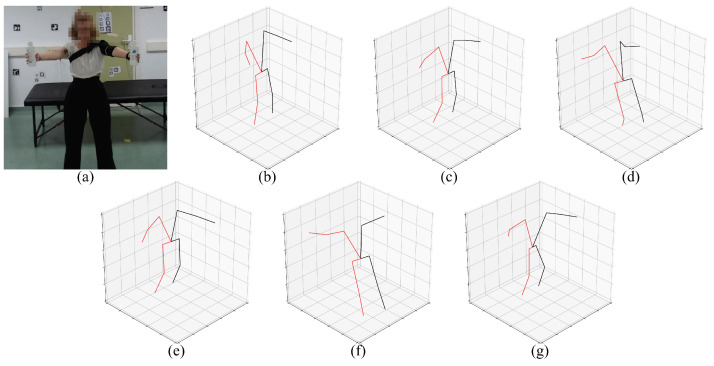
**Preview of the evaluated 3D pose estimation models.** (**a**) Input frame, (**b**) MediaPipe, (**c**) HMR, (**d**) VideoPose3D, (**e**) HybrIK, (**f**) StridedTransformer-Pose3D, (**g**) PoseBERT.

In Table 2, we summarize the selected body pose estimators. The configuration used for each method is detailed in Section 4.3. The methods are classified into two groups: 2D pose estimation methods (body joint detectors, see Figure 4) and 3D pose estimation methods (see Figure 5). Some methods initially perform 2D joint detection to infer from it the full 3D pose; those methods appear in both sections, each referring to the corresponding stage of the processing pipeline. We included the column ‘FPS’ (frames per second) to estimate each method’s speed, given their public implementations. We refer the reader to Section 4.3 for further details about the computation of the FPS.

### 3.1. AlphaPose

AlphaPose [15] uses convolutional neural networks (CNN) to estimate the position of human body parts on an input image or video. It uses a bottom-up approach: first, it locates parts of the body and from them, calculates the orientation and position of the rest of the human body.

Single-person pose estimators are very susceptible to location errors and occlusions since they have been specifically trained with images of a single person. The bottom-up approach provides AlphaPose with a high degree of robustness with respect to the localization of multiple individuals in the same scene and against occlusions or unnatural poses.

Experiments were run on Halpe-FullBody, COCO-WholeBody, COCO and PoseTrack datasets.

### 3.2. MediaPipe

MediaPipe [16] is a framework that groups different inference models and processing algorithms for different input data (images and video). Among these models, the component dedicated to human pose estimation is called BlazePose [23]. BlazePose is a lightweight CNN architecture focused on its use on mobile devices.

The authors employ a pose detector and a tracker configuration. The detector is focused on the location of the individual’s face, from which they can estimate the rest of the body by taking the bounding box of the face as a reference and assuming that the face will always be visible.

Consequently, BlazePose achieves a very light pose estimation system but is strongly linked to the visibility of the individual’s face.

### 3.3. Human Mesh Recovery (HMR)

Related to human pose recovery, there is the problem of shape recovery, which aims to recover the full 3D body shape from the input material; doing so should provide more precise information than tackling the 3D pose alone.

HMR by Kanazawa et al. [17] aims to reconstruct a full 3D mesh of a person by inferring SMPL (Skinned Multi-Person Lineal model) [24] adjustment parameters from an RGB image, those who minimize the joint reprojection error. This is achieved by predicting and iteratively refining camera and SMPL pose and shape parameters, which are later sent to a discriminator model to determine if those parameters codify a valid human shape and pose. As a byproduct, both 3D joint pose and 2D joint projections can be obtained. Experiments were run on LSP, LSP-extended, MPII and MS COCO in-the-wild datasets.

As an advantage of this method, the authors highlight its capability to obtain a 3D pose without relying on an intermediate 2D joint pose location step.

### 3.4. VideoPose3D

Pavllo et al. [18] propose a method to exploit temporal information over 2D pose sequences to predict 3D human pose from video.

In order to do so, they propose an architecture that, using dilated temporal convolutions over sequences of 2D predicted keypoints, can predict 3D poses, being able to capture 2D trajectory information. The training aims to minimize the 2D joint reprojection error obtained after projecting the 3D pose back to the 2D plane and comparing the joint positions with the original 2D keypoints. The 2D keypoints used as input can be obtained using any 2D pose estimation method. Experiments were run using COCO, MPII, Human3.6M and HumanEva-I datasets.

The benefit of this method is twofold: on the one hand, the method is more efficient at estimating 3D pose from 2D keypoints than RNN-based methods, as convolutions allow for the parallel processing of multiple frames. On the other hand, unsupervised training over 2D keypoints dramatically increases the available source material. This is a 3D pose estimation method, but on our comparatives, we also included the performance metrics for the 2D pose estimator used at its base.

### 3.5. KAPAO

A common approach to pose estimation is the joint location through heatmaps. However, those methods suffer from multiple problems, such as the requirement of high-resolution heatmaps, precision loss due to overlapping signals and poor computational efficiency due to post-processing requirements.

To solve some of those limitations, McNally et al. [19] propose KAPAO, a single-stage bounding-box-based 2D pose estimation method. This method works by simultaneously detecting keypoint objects and pose objects: the former represent candidate keypoints, while the latter represent spatial relationships between them. By fusing the detection of both kinds, the model can perform human pose predictions. Models were trained on “COCO train2017” and evaluated on COCO and CrowdPose datasets.

As an advantage of this method, the inference speed is greatly improved. Furthermore, due to its nature, multi-person pose estimation is also possible.

### 3.6. HybrIK

Similarly to HMR, Li et al. [20] aim for joint human pose and shape recovery; the main differentiating factor is the approach to pose recovery, decomposing joint rotations in two categories, twist and swing.

In this method, 3D joints are directly regressed in the form of heatmaps through the use of deconvolution layers (swing angles are implicitly estimated through joint position). At the same time, fully connected layers are used to predict SMPL shape parameters and twist angles (in-plane joint rotations). After obtaining the global root rotation, the final pose and shape parameters can be obtained from the predicted values. Experiments were run on 3DPW, Human3.6M and MPI-INF-3DHP datasets.

Through this method, the authors claim an improved accuracy over pure 3D keypoint-based pose estimation methods and a better reconstruction of the human body mesh.

### 3.7. StridedTransformer-Pose3D

In a similar fashion to VideoPose3D, the authors in [21] aim to predict 3D pose from 2D keypoints sequences; however, they propose a Transformer-based architecture to exploit long-range dependencies.

First, the 2D pose sequence is processed using a Vanilla Transformer Encoder (VTE), which aggregates information from the input 2D sequence to obtain an initial 3D pose sequence. Then, this sequence is processed using a Strided Transformer Encoder (STE), which uses strided convolutions to progressively reduce the sequence length until only the representation of the target pose remains. By doing so, the model can aggregate a long sequence of data into a single frame. Temporal smoothness is enforced through supervision at the output of both the VTE and the STE. Experiments were run on Human3.6M and HumanEva-I datasets.

The advantages of this method are similar to those of VideoPose3D, but the output should be smoother and more precise. We also compared the performance of its base 2D pose estimator.

### 3.8. PoseBERT

Baradel et al. [22] propose a method for human pose estimation training over MoCap data instead of images or videos. This method takes as input a sequence of human poses, which may contain noise, occlusions or even missing frames. Through the use of a Transformer-based architecture, it outputs a sequence of clean and temporally coherent poses. Thus, this method could be described as a pose refinement method, capable of taking the output of any image-based 3D pose estimation method and extending it to leverage temporal information (e.g., from video sources). Models were trained on AMASS MoCap data and tested on 3DPW, MPI-INF-3DHP, MuPoTS-3D and AIST datasets.

We reviewed one of the combinations proposed by the authors, using PoseBERT to process outputs from their MoCap-SPIN model. However, it should be noted that performance may vary when combined with other 3D pose estimation methods.

## 4. Experimental Setup

This section describes the experiments performed with the selected state-of-the-art models explained in the previous section. These experiments were carried out with their default configurations as stated by the authors of the models. First, we describe the experiments carried out in Section 4.1, then in Section 4.2, we explain the metrics applied in order to evaluate our experiments. Finally, in Section 4.3, we describe the technical and software considerations for the analysis of the proposed experiments.

### 4.1. Experiments

**Figure 6:**
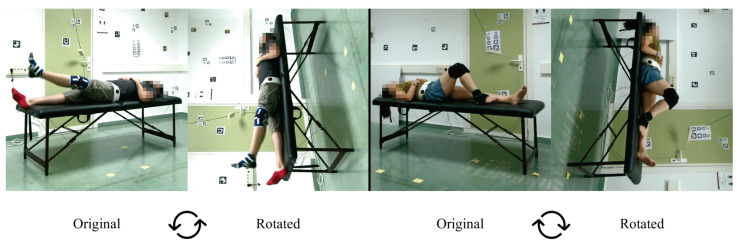
**Original videos vs. Rotated.** Example of exercises 3 (**left**) and 8 (**right**) in supine position (*Original*) and its rotated version (*Rotated*). The experimental results show that several methods perform poorly for the supine posture.

The experiments we carried out are as follows. First, during the early experiments, we noticed that some models were not able to estimate the coordinates of the joints in many frames of the exercises where the position of the subject was supine (exercises 3, 4, 7 and 8). In order to solve this, we decided to rotate these videos by 90clockwise or counter-clockwise in order to simulate that the person is in the standing position (see Figure 6). The results of these experiments are analyzed in Section 5.1.

Second, we compared the performance of the evaluated 2D and 3D pose estimation methods over different groups of exercises. We compared the methods over three different metrics (when applicable): 2D detected keypoints position, joint angles over 2D pose estimations, and joint angles over 3D pose estimations. We considered three groups of exercises, according to the general full-body position: supine, seated and standing (see Table 1 for reference). Trying to make the comparison as fair as possible, for each combination of the group of methods and a group of exercises, we considered only those frames for which we have ground truth values (i.e., OptiTrack was able to provide valid joint positions) and every method delivered a valid estimation (i.e., no empty frames or frames in which the method was not able to detect people). For each case, we performed a Friedman test [25] over the group of methods, ranking the models over each individual frame. If the test detected a statistically significant difference between the groups of values, a Nemenyi post hoc test [26] was performed to find out the differences between the pairs of methods. For ease of interpretation, the results were summarized using critical difference (CD) diagrams [27]. CD diagrams allow to present graphically the result of the comparison between multiple methods, showing the average rank obtained for each one and the range interval from which two methods can be considered different (critical difference, on the top left corner of the diagram); methods on the left side perform better than methods on the right side of the axis, and methods that can be considered equivalent appear connected by a horizontal line. The results per group are presented in Section 5.2.

Third, Section 5.3 analyzes the results by each camera to compare how its angle influences the results of each model over the HPE. As mentioned in Section 2, the dataset was recorded using five different RGB cameras in different positions and angles (see Figure 3 for reference). For this comparison, we obtained the error for the 2D keypoints and for the 2D and 3D angles estimation. This comparison was supported by the Analysis of Variance (ANOVA) statistical test. In the test performed, if the resulting *p*-value was below the significance level of 0.05, the null hypothesis was rejected, indicating that there are significant differences among the group, in this case, between the error of the estimation for each camera viewpoint.

Finally, Section 5.4 compares the ground truth 3D angles of the target limbs, calculated from the 3D keypoints returned by OptiTrack, with respect to the 2D angles calculated from the 2D keypoints returned by the models. The goal of this comparison is to investigate in which situations the estimation of the 2D angles could be comparable to the angles returned by an ideal 3D model if it is the case. For this purpose, we computed the error obtained by the 2D estimators against the 3D ground truth.

It should be noted that only the angles obtained in some lower body exercises (where there is an expected flexion) were used in this study. Specifically, exercises 01, 02, 04, 05, 06 and 08 (see Table 1) were selected.

### 4.2. Metrics

We performed three types of comparisons in order to evaluate the pose estimation methods with respect to the ground truth provided by the IR motion capture system.

The three comparisons made for each frame are shown below, along with the metric used to perform each of them:

1. The error of each method in estimating the 2D coordinates of the three joints to be evaluated.
  For each body joint, we calculated **the Euclidean distance** (see Equation (1)) between the estimated keypoints and the ground truth keypoints. The smaller the distance, the better the estimation of the joint with respect to the ground truth.
  The Euclidean distance *d* between two points, $p=(x_{1},y_{1})$ and $q=(x_{2},y_{2})$, is given by the following equation:
  (1) $$d\left(p,q\right)=\sqrt{{\left(q_{1}-p_{1}\right)}^{2}+{\left(q_{2}-p_{2}\right)}^{2}}$$
  where *p* and *q* correspond to the coordinates of the two points to be compared.
  Finally, the error obtained in each frame during the 2D coordinate estimation is defined as the average of the three Euclidean distances obtained (one per joint).
2. The difference between the bending angle calculated with the estimated 2D coordinates and the one calculated with the ground truth coordinates.
  For each frame, we calculated the bending angle (see Equation (2)) formed by the 2D coordinates of the three joints to be evaluated. We applied the **Mean Absolute Error (MAE)** (see Equation (3)) in order to compare the angle formed with the estimated coordinates and the one formed with the ground truth coordinates:
  (2) $$\alpha =\arccos\left(\frac{\vec{AB}\cdot \vec{AC}}{|\vec{AB}\left|\cdot \right|\vec{AC}|}\right)$$
  where *A*, *B* and *C* are the points involved in the joint.
  The Mean Absolute Error (MAE) between two values is given by the following equation:
  (3) $$MAE\left(\alpha_{1},\alpha_{2}\right)=\frac{1}{N}\sum^{N}|\alpha_{1}-\alpha_{2}|,$$
  where $\alpha_{1}$ and $\alpha_{2}$ correspond to the values of the angles defined by the target joints using the estimated coordinates and the corresponding angles for the ground truth coordinates of each frame, respectively. The parameter *N* represents the total number of processed frames.
3. The difference between the bending angle calculated with the estimated 3D coordinates and the one calculated with the ground truth coordinates.
  For this error, we have applied the same procedure and metrics (**Mean Absolute Error**) as in the previous point. However, in this case, we calculated the bending angle with the 3D coordinates of the three joints instead of the 2D coordinates.

### 4.3. Implementation Details

We developed a small set of software tools in order to perform the analysis presented in this work. We developed a wrapper script for each analyzed method, providing a uniform way of applying the different methods. Each script contains the code required to evaluate the model over an input video and is based on the provided examples and demos contained in its method repository (see Table 3). To the best of our knowledge, no parameters were modified, and the behavior of the methods was not altered in any significant way. Each script also allows storing the pose information in a common format and, optionally, rendering it for visual inspection (see Figure 4 and Figure 5).

For evaluation purposes, all point coordinates were normalized according to the length of the treatment couch (in pixels) in each video. In this way, the comparison between the Euclidean distances is less dependent on some factors such as the proximity of the person to the camera, the resolution of the frames, camera orientations, etc.

In order to measure the methods’ throughput (in FPS), we used the corresponding method implementations (see Table 3). For each method, we processed a single video, with a resolution of 1280×720 pixels, with a length of 44,634 frames (roughly 30 min at 25 FPS, obtained by looping one of the videos of our dataset), and recorded the processing time. We obtained the estimated FPS measurement by dividing the total computation time by the number of frames. By using a long video, we aim to minimize the impact of overhead and other inefficiencies (time spent loading models, accessing the disk, host-GPU communication, etc.). These values are reported in Table 2. The relevant specifications of the machine in which we were running the experiments are the following:

- CPU: Intel(R) Core(TM) i7-11700F (8 cores/16 threads) @ 2.50 GHz (max. freq. 4.90 GHz).
- RAM: 64 GB DDR4 @ 3200 MHz.
- GPU: NVIDIA GeForce RTX 3090 (24 GB VRAM).

## 5. Results

### 5.1. Comparison of Models Using Rotated and Unrotated Videos

Table 4 shows a comparison between the mean errors with confidence intervals at 95% (*p*-value = 0.05) obtained when estimating the 2D coordinates of the joints (2D keypoints) and the degrees of flexion (2D–3D) using the original videos (**Original**) and the rotated ones (**Rotated**). The distance (in pixels) between estimated coordinates and ground truth was normalized to the projected length of the treatment couch (also in pixels), so an error of 1 would mean that the error is the same length as the couch itself.

In this way, we can see how the person’s posture influences the detection and estimation of their joints.

If we look at Table 4, we can see that, in most cases, the rotated videos obtain better results (i.e., lower error) both in the mean and confidence interval at 95% (green cells with bold font).

There are some exceptions, such as MediaPipe, in which the error in detecting the 2D coordinates improves when rotating the videos. At the same time, it remains similar in the estimation of the 2D and 3D degrees (yellow cells). This may be because the improvement experienced by this model in the estimation of the keypoints 2D is small (0.001) and, therefore, does not influence the estimation of the angle much.

In the case of VideoPose3D, if we use the rotated videos, the error in the estimation of 2D keypoints and joint angles decreases. At the same time, it increases in the estimation of 3D joint angles (red cells and italic font). If we look at Figure 6, which shows an example of a rotated video, we can see that the scene and the person’s posture are unnatural. This may cause some difficulties with obtaining 3D keypoints.

Thus, we can see that the person’s posture influences the detection in the image of the target body joints since, in general, most of the models obtain better results with rotated videos. This leads us to suppose that the models were trained much more for detecting people in a more typical position (standing position) than in other positions, such as the supine position.

Since we obtained a lower error with the estimations made on the rotated videos, the rest of the comparisons shown in the article were made considering the rotated videos instead of the original ones.

### 5.2. Model Comparison

The results per group are presented below.

#### 5.2.1. Supine (Rotated)

**Figure 7:**
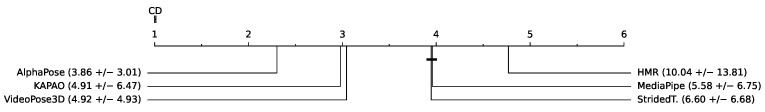
**Ranking of methods by 2D keypoints estimation error, supine position.** In pixels, normalized with respect to the projected length of the treatment couch (values appear multiplied × 100).

**Figure 8:**
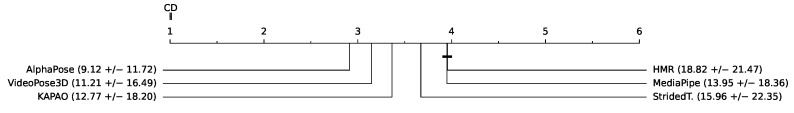
**Ranking of methods by 2D joint angle estimation error, supine position.** Shown in degrees.

**Figure 9:**
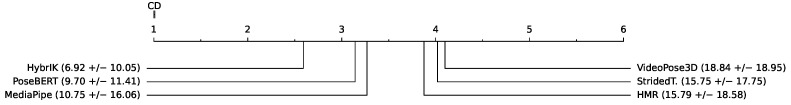
**Ranking of methods by 3D joint angle estimation error, supine position.** Shown in degrees.

The exercises here comprise the leg joints (ankle, knee and hip). As seen in Figure 7, the method that performs the best in the 2D keypoint detection phase (in rotated supine position) is AlphaPose, with a mean error of 0.0386 ± 0.0301 pixels (normalized with respect to the length of the treatment couch). Also, there is a tie between MediaPipe and StridedTransformer-Pose3D (no statistically significant difference has been found between their results). It is similar in the case of 2D joint angle estimation error (see Figure 8), as 2D joint angle estimation can be considered as equivalent to 2D keypoint estimation. As it is computed in this work, for a given set of three identified keypoints (i.e., we know to which joint they correspond), they can only form one single angle and thus, the joint angle error only depends on the accuracy of the keypoint estimations. Notice how both rankings mostly agree (there is a discrepancy between KAPAO and VideoPose3D, but they keep consecutive positions; also now, there is a match between HMR and MediaPipe). In the case of 3D joint angle estimation error (see Figure 9), the best method seems to be HybrIK, with a mean error of 6.92 ± 10.05 degrees.

- 2D keypoints:

- 2D joint angles:

- 3D joint angles:

#### 5.2.2. Seated

**Figure 10:**
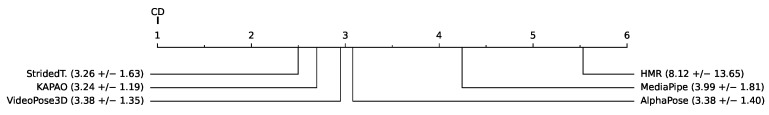
**Ranking of methods by 2D keypoint estimation error, seated position.** Shown in pixels, normalized with respect to the projected length of the treatment couch (values appear multiplied × 100).

**Figure 11:**
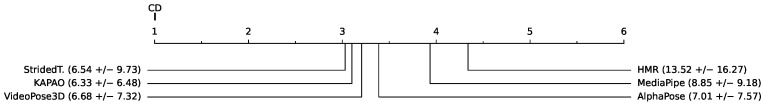
**Ranking of methods by 2D joint angle estimation error, seated position.** Shown in degrees.

**Figure 12:**
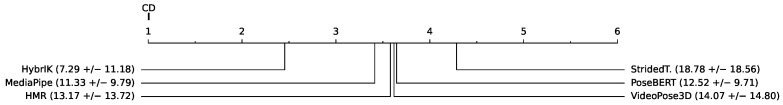
**Ranking of methods by 3D joint angle estimation error, seated position.** Shown in degrees.

Exercises here mostly comprise the joints of the leg, with the exception of the circular pendulum (see Table 1). As seen in Figure 10, the method which performs the best when detecting 2D keypoints (in seated position) is StridedTransformer-Pose3D, with a mean error of 0.0326 ± 0.0163 pixels (normalized with respect to the length of the treatment couch); there is a perfect agreement with the 2D joint angle estimation error ranking (see Figure 11). In the case of 3D joint angle estimation, the best-performing method is HybrIK, with a mean error of 7.29 ± 11.18 degrees (see Figure 12).

- 2D keypoints:

- 2D joint angles:

- 3D joint angles:

#### 5.2.3. Standing

**Figure 13:**
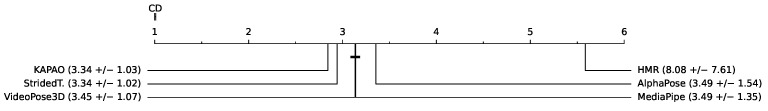
**Ranking of methods by 2D keypoints estimation error, standing position.** Shown in pixels, normalized with respect to the projected length of the treatment couch (values appear multiplied × 100).

**Figure 14:**
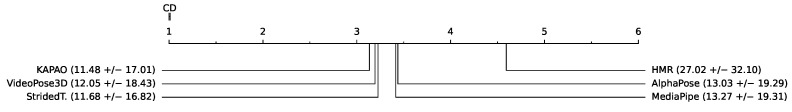
**Ranking of methods by 2D joint angle estimation error, standing position.** Shown in degrees.

**Figure 15:**
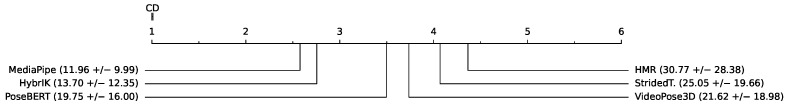
**Ranking of methods by 3D joint angle estimation error, standing position.** Shown in degrees.

Exercises here comprise the joints of the arm (wrist, elbow and shoulder). In Figure 13, we can see how the best method, when performing 2D keypoint estimation, is KAPAO, with a mean error of 0.0334 ± 0.0103 pixels (normalized with respect to the length of the treatment couch). Also, notice how (in this task) there is no statistically significant difference between VideoPose3D and MediaPipe. Results mostly agree with the ranking by 2D joint angle estimation error (see Figure 14); two consecutive methods switch their positions, StridedTransformer-Pose3D and VideoPose3D, but the rest of the methods occupy the same positions as in Figure 13. Regarding the 3D joint angle estimation error (see Figure 15), the best performer is MediaPipe with a mean error of 11.96 ± 9.99 degrees, followed by HybrIK with a mean error of 13.70 ± 12.35 degrees.

- 2D keypoints:

- 2D joint angles:

- 3D joint angles:

### 5.3. Results per Camera Viewpoint

Table 5, Table 6 and Table 7 show the mean error for the estimation of 2D keypoints and for the estimation of angles in 2D and 3D, respectively. This average was considered among all the exercises carried out.

As we can see from the above-mentioned tables, the results indicate that the best camera viewpoint for most models is cam0 and cam1, which correspond to the most centered and frontal viewpoints in our configuration (see Figure 3 for reference). Regarding the ANOVA test, the results for the hypothesis test with the data in Table 5, Table 6 and Table 7 were practically *p*-value = 0.0, which means that there are significant differences between the mean errors achieved by the models and the camera’s point of view. In all cases, Tukey’s test [28] showed significant differences between the cameras.

**Figure 16:**
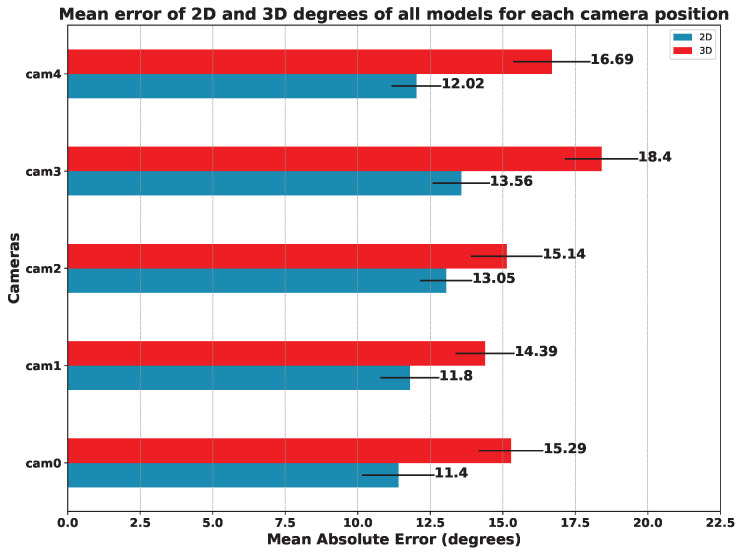
**Mean Absolute Error on the estimated angles (in degrees).** The MAE was computed for each model and camera position: the lower, the better.

**Figure 17:**
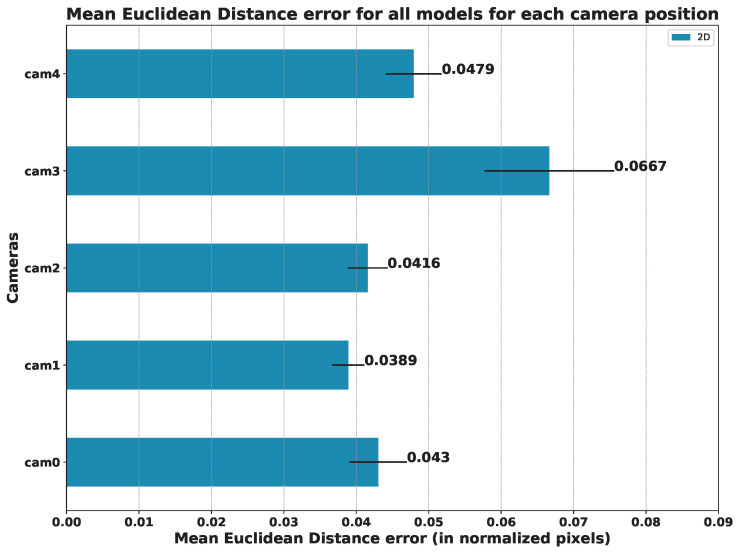
**Mean Euclidean Distance for all models for each camera position** (in normalized pixels): the lower, the better.

In order to compare the influence of the viewpoint of the camera in relation to the patient, we also compared the mean error of all models on each camera. The results of this study are summarized in Figure 16 (angles) and Figure 17 (keypoints).

Focusing on the position of the cameras, we can see that the one that offers the best results in terms of the mean error of all the models is camera 1, followed by camera 0. It is to be expected that the cameras with a more frontal viewpoint are the ones that obtain better results since the models used were trained with datasets whose images are not usually captured from too-steep angles. This makes models sensitive to error when images are obtained from more open angles, as is the case for cameras 3 and 4 (see Figure 3).

### 5.4. Comparison between the 3D Angles of the Ground Truth with Respect to the 2D Angles of the Models

Table 8 shows, for each 2D model, the Mean Absolute Error obtained by comparing the 3D degrees of the ground truth with respect to the 2D angles of these models. In addition, it also shows the mean error of each model obtained in the five cameras and the mean error of each camera, taking into account all the 2D models. Similarly to the previous tables, in this case, we applied an ANOVA test to determine the impact of the camera viewpoint on the results. The findings were consistent with the previous tests, revealing that the camera viewpoint is a crucial factor in terms of the error returned by the pose estimation models.

It should be noted that both the model used and the camera orientation considerably influence the estimation error. On the one hand, AlphaPose is the model with the lowest error in terms of 2D angles and, therefore, it is the best choice to consider if we only want to use 2D information. On the other hand, camera 1 and camera 0 have the lowest estimation error, as they have a more frontal view of the exercise, while camera 3 and camera 4, which are located on the sides, show the highest error. Thus, using the cameras that show a frontal view of the exercise is another aspect to consider if we only want to use the 2D estimators with the smallest error. Reference values for the measurement of limb angles can be found in [29], where the authors establish a Root Mean Square Error (RMSE) of 4.95 with (1.24–7.04) standard deviation for knee flexion using Inertial Measurement Units (IMUs) sensors.

**Figure 18:**
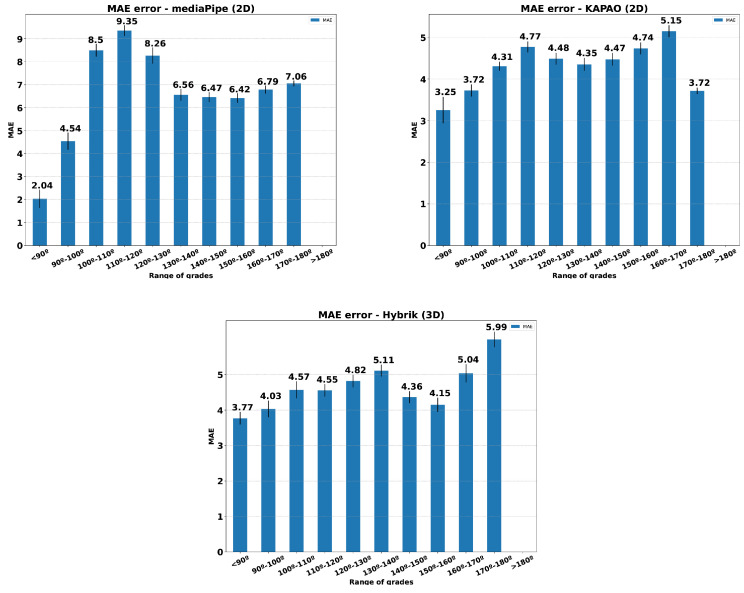
**Mean Absolute Error on the estimated angles (in degrees).** The MAE was computed for each model and camera position: the lower, the better.

Figure 18 presents histograms relating the MAE for three methods to the angle opening in the performance of knee flexion exercise. The MAE was calculated using all the individuals in the dataset and both view sides. Only Exercise 1 was considered. This comparison aims to determine whether the estimation methods are sensitive to the aperture angle during the exercise. As we can see, the error distribution remains below the reference values set by [29] for the HybrIK and KAPAO methods.

## 6. Discussion

The results presented in the previous section indicate that the individual’s posture and the camera’s position and angle are relevant for effective pose estimation. The exercises in which the patient is lying down were especially challenging for the pose estimation models. However, we found that rotating these videos can lead to better results. Almost all models improved their results with respect to body joint estimation accuracy or the detection of individuals in the scene in rotated videos over unrotated ones (see Section 5.1).

The recorded dataset can be very useful for future research, as not many public datasets are dedicated to the physical rehabilitation of patients with musculoskeletal impairments. In [11], the authors provide 3D skeleton joint positions for 29 individuals with annotations about the exercises and the performance quality (correct or not). In [30], the authors also provide the position of the joints for upper-limb exercises of 19 participants and clinical assessment scores annotated by experts. None of the above-mentioned datasets includes video information that can be used for a human pose estimation approach.

With regard to 2D and 3D pose estimation, we found that specific models stand out differently from others depending on the patient’s position. Specifically, for 2D pose estimation, AlphaPose stands out with better results when the patient’s position is supine (with the video rotated). If the patient’s position is seated, the method that stands out is StridedTransformer-Pose3D and, finally, if the patient is standing, the model with the best results is KAPAO (see Section 5.2).

For 3D pose estimation, HybrIK provides the best results for seated and supine positions. Only for standing, MediaPipe achieves better results (see Section 5.2). However, it should be noted that the use of MediaPipe is more straightforward and comes ready to be integrated into projects with Android, iOS or Javascript for mobile devices.

In the study carried out concerning the position of the camera, the methods that undoubtedly stand out are AlphaPose for 2D estimation and HybrIK for 3D estimation. Focusing on the mean error of all the models, the camera that provided the best results is camera 1, which has a top–center position with respect to the subject. The errors increase proportionally as the angle from which the scene is recorded becomes more open (see Section 5.3).

Finally, when comparing the ground truth 3D degrees with respect to the 2D degrees obtained by the models, we find a Mean Absolute Error that varies between 7 and 14 degrees of difference, being of great influence on both the selected 2D model and the orientation of the cameras. Specifically, in the case of wanting to use only the 2D information instead of the 3D, considering the error that would be committed, it would be ideal to use the AlphaPose model since it is the one that obtains the lowest average error in the five cameras. Meanwhile, concerning the cameras, it would be convenient to use the front cameras, specifically camera 1, instead of the side cameras since, as in previous comparisons, those are the cameras that return a lower error in the estimation (see Section 5.4).

Regarding the possible limitations of this study, we should mention the following considerations.

Obtaining ground truth data for the target rehabilitation exercises by using photogrammetry requires fulfilling the working conditions of the OptiTrack system. This includes the correct and fixed positioning of the infrared sensors and controlled illumination conditions. As this might be considered a limitation regarding the variety of recording scenarios, note that physical rehabilitation is mostly performed indoors in controlled environments.

However, as indicated in Section 2, the OptiTrack suffers from some shortcomings regarding the registration of the position of some sensors that are hidden in some frames, mainly due to the movement made by the individual performing the exercises. It is worth noting that our data were recorded in a controlled environment with a consistent background. This may limit the applicability of our study to other scenarios that are not controlled. This limitation is something that needs to be addressed in future work.

The range of movements considered in this dataset version corresponds to the target rehabilitation exercises. For future extensions of the dataset, including additional subjects, it would be interesting to add new movements not only for rehabilitating specific limbs but also for the accurate performance of sports exercises (e.g., fitness or athletics).

**Figure 19:**
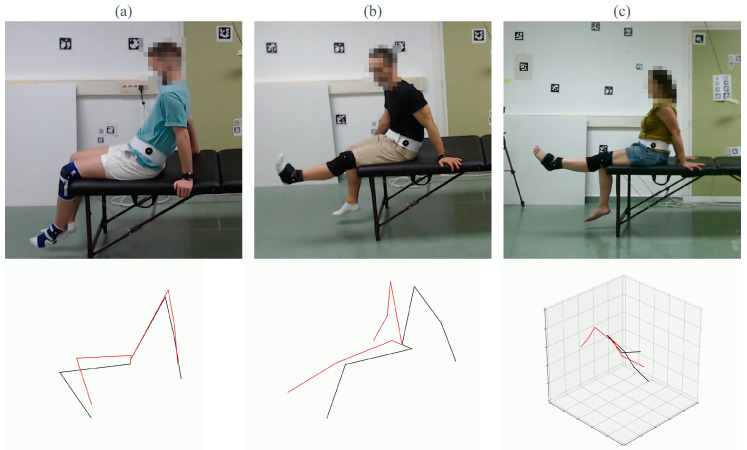
**Limitations of HPE methods.** Examples of pose estimation failures for Mediapipe 2D (**a**), KAPAO (**b**) and HybrIK (**c**). Top row: test frame from the dataset. Bottom row: pose estimation returned by the corresponding method.

On the other hand, the pose estimation methods evaluated in this work are for general purposes, trained with generalist datasets with a large number of data but not specifically for physical rehabilitation. The performance of these methods can be biased by the data used in the training phase, so the particular conditions of our data can lead to bad performance of the method. As observed in Figure 19, even the best-performing methods have several limitations regarding occlusions or erroneous identifications of limbs. Each column of the figure corresponds to one of the best methods we tested throughout this study. The selected frame corresponds to the frame with the highest MAE. The first row shows the frame of the dataset, and the second one is the estimation obtained by the HPE method.

The first column (a) shows the error made by Mediapipe, where the legs of the treatment couch misled the estimation of the ankles. In (b), KAPAO exchanged the estimation of both legs. Finally, (c) shows the wrong estimation of HybrIK, in which the method does not recognize the overall pose correctly.

Although training pose estimators is not the goal of the proposed dataset, we hope that fine tuning the evaluated methods on our dataset could help address those methods’ current limitations.

## 7. Conclusions

We presented an extensive dataset of multiple physical rehabilitation exercises focusing on various limbs and body positions.

Subsequently, eight 2D and 3D pose estimators were selected to estimate the 2D and 3D coordinates of the joints of the studied limbs and to compare them with our ground truth. In this way, we were able to determine how the position of the individual influences the estimation of their joints, as well as which method is more suitable for each situation and from which point of view it should be used.

Overall, for the 2D pose estimations, AlphaPose, StridedTransformer-Pose3D and KAPAO are the methods with the best results for the supine, seated and standing positions, respectively. On the other hand, for the 3D pose estimations, HybrIK provided the best results for the seated and supine positions, while for standing exercises, MediaPipe was the best; its ease of deployment is also a plus for MediaPipe.

In addition, we were also able to determine how the camera angle influenced our results, with the camera that was fully frontal and at the top height obtaining the best results.

Finally, regarding the comparison between 3D and 2D estimation methods, we found that the error when using 2D estimates instead of 3D estimates is highly influenced by the selected model, with AlphaPose being the model with the lowest error in the 2D estimates. It is also influenced by the orientation of the cameras, with the fully frontal and top–center camera being the one which induced the smallest error in almost every 2D model.

The results of the conducted study suggest that Computer Vision methods can be a cost-effective approach to aid the rehabilitation process, encouraging us to continue with this research line. Taking into consideration the obtained results and future implementations of rehabilitation systems, we recommend MediaPipe as the pose estimation method due to the ease of integration with different systems since it is already implemented in multiple platforms such as Android, iOS, Javascript or Python. Moreover, this method simultaneously provides 2D and 3D estimations with quite low errors compared to other methods analyzed in this paper.

In future work, we plan to expand the dataset with new subjects and increase the number of joints measured by OptiTrack. In addition, we will use additional pose techniques, such as PoseBert [22], to try to recover the missing keypoints lost during the recording of the dataset to see if it improves our results. Moreover, it would be interesting to perform fine tuning on the best-performing models of this study and to consider the option of training specific models for particular exercises and body postures (e.g., supine). Also, this study was oriented towards the technical analysis of the feasibility of such vision-based solutions. Given the obtained results, we plan to use these techniques with real patients with in-the-wild settings.

## Figures and Tables

**Table 1:** **List of exercises included in the UCO Physical Rehabilitation dataset.** They are classified by position, side and upper/lower body.

#	Exercise Name	Position	Side	Upper/ Lower Body
01	Bending the knee without support while sitting	Seated	Left	Lower
02	Bending the knee with support while sitting			
03	Lift the extended leg	Supine		
04	Bending the knee with bed support			
05	Bending the knee without support while sitting	Seated	Right	
06	Bending the knee with support while sitting			
07	Lift the extended leg	Supine		
08	Bending the knee with bed support			
09	Shoulder flexion	Seated	Left	Upper
10	Horizontal weighted openings	Standing		
11	External rotation of shoulders with elastic band			
12	Circular pendulum			
13	Shoulder flexion	Seated	Right	
14	Horizontal weighted openings	Standing		
15	External rotation of shoulders with elastic band			
16	Circular pendulum			

**Table 2:** **Summary of the evaluated pose estimation methods**. The “2D” and “3D” columns describe the dimensional capability of the estimation methods. The “Video-based” column shows whether the input for the method is a video or not. The “Num. joints” column shows the number of joints returned by the method. “Kp. detector included” shows whether the method needs an external keypoint detector or is included in the method itself. The “FPS” column shows the frames per second that the method is capable of processing, and finally, the “GPU/CPU” column shows where the method can process the data. (N/A stands for “not applicable”).

Method	2D	3D	Video Based	Num. Joints	Kp. Detector Included	FPS	GPU/CPU
AlphaPose [15]	✓	✗	✓	17	✓	15.93	GPU
MediaPipe [16]	✓	✓	✓	33	✓	66.58	CPU
HMR * [17]	✓	✓	✗	19	✗	18.03	CPU ***
VideoPose3D * [18]	✓	✓	✓	17	✗	19.09	GPU
KAPAO [19]	✓	✗	✗	17	✓	41.43	GPU
HybrIK [20]	✗	✓	✗	17	✓	13.21	GPU
StridedTransformer-Pose3D * [21]	✓	✓	✓	17	✗	21.62	GPU
PoseBERT ** [22]	✗	✓	✓	24	N/A	24.30	GPU

* 3D-from-2D models, 2D corresponding to base 2D detection model. ** Pose refinement model, number of joints corresponding to base 3D pose estimator. *** GPU not used due to a memory leak not present when using CPU.

**Table 3:** **Code repositories of each of the methods used in this work.** The default configuration used can be found in these repositories.

Methods	Implementation
AlphaPose [15]	https://github.com/MVIG-SJTU/AlphaPose (Last access: 19 September 2023)
MediaPipe [16]	https://github.com/google/mediapipe (Last access: 19 September 2023)
HMR [17]	https://github.com/akanazawa/hmr (Last access: 19 September 2023)
VideoPose3D [18]	https://github.com/facebookresearch/VideoPose3D (Last access: 19 September 2023)
KAPAO [19]	https://github.com/wmcnally/kapao (Last access: 19 September 2023)
HybrIK [20]	https://github.com/Jeff-sjtu/HybrIK (Last access: 19 September 2023)
StridedTransformer-Pose3D [21]	https://github.com/Vegetebird/StridedTransformer-Pose3D (Last access: 19 September 2023)
PoseBERT [22]	https://github.com/naver/posebert (Last access: 19 September 2023)

**Table 4:** **Mean error at 95% confidence (*****p*****-value = 0.05)** of each model in the estimation of keypoints (distance in pixels normalized to the projected length of the treatment couch) and degrees of exercises 3, 4, 7 and 8 (with and without rotation). The results shown in the green cells and bold are the cases in which the rotated videos improve on average; the yellow cells show the cases in which there is no improvement on average, but there is improvement in terms of standard deviation. Finally, the red cells and italic font show the cases where there is no improvement.

	2D Keypoints ↓		∡ Degrees 2D ↓		∡ Degrees 3D ↓	
	**Original**	**Rotated**	**Original**	**Rotated**	**Original**	**Rotated**
ine AlphaPose	0.044 ± 0.005	**0.039 ± 0.003**	11.80 ± 2.72	**9.32 ± 1.36**	**-**	**-**
ine MediaPipe	0.051 ± 0.010	**0.050 ± 0.009**	13.43 ± 3.50	14.23 ± 2.47	10.30 ± 2.53	10.76 ± 2.04
ine HMR	0.138 ± 0.049	**0.117 ± 0.018**	23.79 ± 2.51	**19.59 ± 4.01**	20.65 ± 2.45	**15.56 ± 2.90**
ine VideoPose3D	0.074 ± 0.011	**0.053 ± 0.009**	19.71 ± 2.21	**12.08 ± 2.11**	17.81 ± 1.81	*18.17 ± 4.71*
ine KAPAO	0.067 ± 0.011	**0.050 ± 0.007**	11.19 ± 1.49	*13.01 ± 1.89*	**-**	**-**
ine HybrIK	**-**	**-**	**-**	**-**	17.93 ± 2.34	**6.86 ± 1.20**
ine Strided	0.034 ± 0.002	*0.068 ± 0.013*	15.98 ± 6.19	16.66 ± 3.44	19.43 ± 2.92	**15.42 ± 2.86**
ine PoseBERT	**-**	**-**	**-**	**-**	9.87 ± 1.26	**9.62 ± 1.26**
ine						

**Table 5:** **Average keypoint error per camera for each 2D method** (in pixels, normalized with respect to the projected length of the treatment couch). The names StridedTransformer-Pose3D and VideoPose3D are shortened to Strided and VPose3D, respectively, for space reasons. The smallest mean keypoint error per model is marked in bold.

*d* * ↓	Alphapose	Mediapipe	HMR	VPose3D	Kapao	Strided
cam0	0.033 ± 0.0246	0.0403 ± 0.0248	0.0787 ± 0.0715	**0.0346 ± 0.0306**	**0.0297 ± 0.0195**	0.0359 ± 0.0305
cam1	**0.0311 ± 0.0151**	**0.0363 ± 0.015**	**0.0586 ± 0.0446**	0.0351 ± 0.0264	0.0313 ± 0.0311	**0.0355 ± 0.0252**
cam2	0.0337 ± 0.0046	0.0369 ± 0.0163	0.0666 ± 0.0591	0.0371 ± 0.0301	0.0338 ± 0.0463	0.0371 ± 0.0277
cam3	0.041 ± 0.0076	0.0569 ± 0.0662	0.1668 ± 0.2756	0.0514 ± 0.0468	0.0431 ± 0.0411	0.0551 ± 0.0612
cam4	0.0359 ± 0.0062	0.0451 ± 0.0361	0.0855 ± 0.0873	0.0409 ± 0.0321	0.0354 ± 0.0289	0.0428 ± 0.0419

* Euclidean Distance.

**Table 6:** **Average joint angle error per camera for each 2D method** (in degrees). The smallest MAE per model is marked in bold.

MAE * ↓	Alphapose	Mediapipe	HMR	VPose3D	Kapao	Strided
cam0	8.41 ± 12.61	**10.32 ± 13.62**	19.68 ± 25.5	**8.14 ± 11.91**	**7.31 ± 10.11**	**8.62 ± 13.65**
cam1	**8.03 ± 11.76**	10.7 ± 13.21	**17.78 ± 24.49**	9.9 ± 15.04	8.79 ± 14.34	10.14 ± 15.31
cam2	9.67 ± 16.42	11.38 ± 16.71	18.66 ± 23.89	10.49 ± 17.98	9.64 ± 16.77	10.04 ± 16.71
cam3	10.16 ± 14.32	14.03 ± 20.63	20.73 ± 25.22	10.96 ± 16.73	9.87 ± 14.91	12.74 ± 20.84
cam4	9.25 ± 11.67	11.22 ± 14.14	18.2 ± 21.05	9.34 ± 13.31	8.72 ± 12.58	11.23 ± 18.99

* Mean Absolute Error.

**Table 7:** **Average joint angle error per camera for each 3D method** (in degrees). The smallest MAE per model is marked in bold.

MAE * ↓	Mediapipe	HMR	VPose3D	HybrIK	Strided	PoseBERT
cam0	9.99 ± 10.33	19.45 ± 22.37	14.49 ± 15.23	8.99 ± 11.88	**15.16 ± 14.2**	13.31 ± 12.61
cam1	10.36 ± 9.56	**16.08 ± 21.24**	**13.59 ± 14.16**	7.71 ± 10	17.49 ± 17.85	**12.17 ± 12.38**
cam2	**9.03 ± 8.81**	17.57 ± 22.09	16.28 ± 17.51	**7.51 ± 9.6**	17.39 ± 15.56	12.67 ± 13.15
cam3	13.47 ± 17.67	19.48 ± 22.13	21.22 ± 20.22	10.39 ± 14.04	22.2 ± 20.44	14.62 ± 13.78
cam4	11.70 ± 11.77	18.1 ± 18.88	19.43 ± 19.45	8.59 ± 11.69	22.31 ± 24.43	13.09 ± 12.69

* Mean Absolute Error.

**Table 8:** **Comparison between the 3D angles of the ground truth with respect to the 2D angles of the models.** The **Mean Absolute Error** (in degrees) between the 3D angles of the ground truth and the 2D angles of the models is shown for some lower body exercises. The smallest MAE per model is marked in bold.

	Cam0	Cam1	Cam2	Cam3	Cam4	Model AVG
**AlphaPose**	5.69 ± 9.5	5.98 ± 7.32	**5.36 ± 6.13**	8.81 ± 9.9	7.91 ± 9.14	**6.75**
**MediaPipe**	**7.37 ± 8.76**	8.79 ± 7.58	7.48 ± 6.58	12.4 ± 14.87	10.72 ± 10.7	9.36
**HMR**	13.69 ± 16.22	**10.49 ± 12.46**	11.87 ± 14.6	15.84 ± 21.24	17.32 ± 18.28	13.84
**VPose3D**	**6.25 ± 9.53**	9.54 ± 15.59	8.02 ± 14.36	12.52 ± 18.52	9.24 ± 12.76	9.11
**KAPAO**	**5.14 ± 8.47**	8.25 ± 14.78	7.19 ± 13.41	10.44 ± 15.46	8.2 ± 11.65	7.84
**Strided**	**6.12 ± 9.13**	10.92 ± 17.42	7.94 ± 13.5	12.22 ± 19.57	12.22 ± 22.49	9.88
**Camera AVG**	**7.38**	8.99	9.57	12.04	10.94	

## Data Availability

Dataset available at https://github.com/AVAuco/ucophyrehab under request (Last access: 19 September 2023).

## Abbreviations

The following abbreviations are used in this manuscript:

HPE	Human Pose Estimation
IR	Infrared
FPS	Frames per second
HMR	Human Mesh Recovery
SMPL	Skinned Multi-Person Lineal
RNN	Recurrent Neural Network
VTE	Vanilla Transformer Encoder
STE	Strided Transformer Encoder
MAE	Mean Absolute Error
RMSE	Root Mean Square Error
